# Effects of Monochromatic and Composite Light Withering on Black Tea Aroma

**DOI:** 10.3390/foods14132232

**Published:** 2025-06-25

**Authors:** Yafang Li, Bilin Li, Ziyan Zhu, Wushuang Zhang, Jingwen Yang, Wei Xu, Ling Lin

**Affiliations:** 1 College of Horticulture, Sichuan Agricultural University, Chengdu 611130, China; liyafang@stu.sicau.edu.cn (Y.L.); libilin1005@126.com (B.L.); 18383138385@163.com (Z.Z.); zhangwushuang@stu.sicau.edu.cn (W.Z.); yangjingwen1@stu.sicau.edu.cn (J.Y.); 2 Tea Resources Utilization and Quality Testing Key Laboratory of Sichuan Province, Chengdu 611130, China

**Keywords:** black tea, withering, aroma, monochromatic light, composite light

## Abstract

In this study, the effects of different monochromatic (red, blue, and yellow light) and composite (red–blue and red–yellow) LED light withering on the aroma of black tea was investigated. The results showed that among monochromatic LED treatments, red light withering achieved the highest sensory evaluation score for aroma. However, yellow light withering enhanced soluble sugar content and reduced tea polyphenol levels. It also increased the total amount of volatile compounds more effectively than red or blue light treatments. Nevertheless, single-wavelength LED withering was less effective than natural light in aroma improvement. In contrast, composite light withering outperformed single-wavelength LED treatments in improving black tea aroma, with the red–yellow light combination being more pronounced. It elevated the level of hydrocarbons, certain aldehydes, and alcohols, which ultimately impart an almond-like and roasted aroma profile to the black tea. The findings suggested that appropriate composite light withering can effectively improve the aroma of black tea.

## 1. Introduction

Black tea, the most widely consumed tea globally, is highly valued for its distinctive and charming flavor [1]. The processing of black tea includes withering, rolling, fermentation, and drying [2]. Of these, withering is the initial step in the processing of black tea and a key stage that affects the quality of black tea [3,4]. During withering, fresh tea leaves diminished moisture under controlled temperature and humidity [5]. Simultaneously, macromolecular compounds in the tea leaves are hydrolyzed and the flavor of tea develops with the changes in volatile compounds [6,7,8].

The formation of black tea aroma is a complex process influenced by multiple factors, which involves all stages of black tea processing [9]. Withering is a crucial process that affects the aroma of black tea [10,11]. In this process, low-boiling-point aroma substances such as cis-3-Hexen-1-ol gradually decrease, while both the variety and the total content of small-molecule volatiles like alcohols and aldehydes enhance with the increase in hydrolytic enzyme activity [3]. These compounds mainly present a floral and fruity fragrance. Studies have found that withering method, as well as parameters like withering time, temperature, humidity, and light quality, can affect the aroma of black tea [2,12,13].

Light is an important factor that influences the metabolism of tea leaves, and similarly, light quality during withering can easily affect the volatile profile of black tea [14]. It was found that red light withering improved the floral and sweet aroma of black tea and white tea while strengthening the chestnut-like fragrance of green tea [15,16]. Blue and red light withering reduced the grassy aroma and enhanced floral–fruity characteristics [14]. In addition, yellow light withering enhanced the fruity aroma of tea by promoting terpenoid accumulation [17]. These results indicate that monochromatic LED light withering is effective in improving both the composition and content of volatile components. Notably, sunlight withering also improves the flavor of tea [3] and is widely applied in production. In this method, tea leaves were exposed to sunlight to enhance the floral and fruity aroma by spurring non-enzymatic reactions, enhancing the activity of related enzymes, or up-regulating the expression of these enzymes [18]. It is well known that sunlight contains a lot of visible and invisible light. Based on this, we hypothesized that the mixture of different monochromatic lights could also improve the aroma of tea. However, little research has been performed on the effect of mixed light on the aroma of black tea.

Therefore, in this study, different monochromatic lights (red, blue, and yellow light) and composite light (red–blue and red–yellow composite light) were applied in the withering of black tea, and the effects of withering with different light qualities on the volatile components of black tea were investigated by using sensory evaluation and headspace solid-phase microextraction combined with gas chromatography–mass spectrometry (HS-SPME-GC-MS). The findings will provide a theoretical foundation and technical guidance for optimizing exogenous light withering in black tea processing and improving black tea quality.

## 2. Materials and Methods

### 2.1. Chemical Regeants

Folin and Ciocalteu’s phenol reagent was purchased from Titan Technology Co., Ltd. (Shanghai, China). Sodium carbonate, disodium hydrogen phosphate, anthrone, ninhydrin, methanol, and sodium chloride were from Sinopharm Chemical Reagent Co., Ltd. (Beijing, China). Chromatographic grade C7–C40 n-alkane mixture was obtained from Merck (Darmstadt, Germany). Chromatographic-grade ethyl caprate was purchased from Sigma-Aldrich (Darmstadt, Germany).

### 2.2. Preparation of Tea Samples

Fresh tea leaves (one bud and two leaves) of ‘Fuding Dabai’ (*Camellia sinensis* (L.) O. Ktze) were harvested in Qionglai, China, on July 2024. The tea leaves were subjected to five different withering treatments: red light withering (RL, 605~700 nm, 21.6~125.1 lx), blue light withering (BL, 450~480 nm, 20.1~161 lx), yellow light withering (YL, 580~595 nm, 1.7~145.4 lx), red–blue composite light (RB, 1:1 ratio, red light, 620~660 nm; blue light, 450~470 nm; 25.6~130 lx), red–yellow composite light (RY. 1:1 ratio, red light, 620~680 nm; yellow light, 580~610 nm; 25~160 lx), and natural light withering (CK).

All treatments were withered under controlled conditions (29–30 °C, 60% relative humidity) for 7 h with a leaf layer thickness of approximately 5–6 cm. During this period, the leaves were turned every 3 h. Subsequently, the leaves were rolled by a rolling machine (6CR-25, Zhejiang Zhufeng Machinery Co., Ltd., Quzhou, China) for 40 min at room temperature (29–30 °C). The rolled leaves were then fermented at 30 °C for 4 h (90% relative humidity) by using a fermentation chamber (6CFJ-1B, Fujian jiayou, Fuzhou, China). Finally, the leaves were dried (first at 120 °C for 20 min, followed by 80 °C for 1 h) in a drying oven (JY 6CHZ-1B, Fujian jiayou). Three replicates were carried out, and about 5 kg of fresh leaves was used per batch.

### 2.3. Sensory Evaluation

A sensory evaluation of the samples was conducted strictly in accordance with the Chinese National Standard ‘Methodology for sensory evaluation of tea’ (GB/T 23776-2018) [19] and was approved by the Ethnic Committee of Sichuan Agricultural University. All participants were fully informed of the research requirements and potential risks and voluntarily participated in the sensory evaluation.

Specifically, 3 g of black tea was infused in boiling drinking water (100 °C) for 5 min. Then, the tea infusion was separated from the leaves for evaluation. Five trained professionals independently evaluated and scored each samples in terms of appearance, aroma, color, taste, and infused leaf, subsequently. The average score of the five professionals was the final score for the sample.

### 2.4. Determination of Major Quality Components

The content of moisture and water extracts was measured according to the Chinese National standards GB 5009.3-2016 [20] and GB/T 8305-2013 [21], respectively. The tea polyphenols (TP) content was determined by Folin and Ciocalteu’s phenol reagent (GB/T 8313-2018) [22] using an ultraviolet–visible spectrophotometer (UV2300, Jinghong, Shanghai). Free amino acid (FAA) content was measured by using the ninhydrin method (GB/T 8314-2013) [23]. Total soluble sugar content was determined with an anthrone reagent at 620 nm after extraction with 80 % (*v*/*v*) ethyl alcohol solution at 50 °C for 20 min [24].

### 2.5. Identification of Volatile Compounds

#### 2.5.1. Extraction of Volatile Compounds by HS-SPME

The extraction of volatile compounds was performed according to the method described in our previous study [25] with slight modifications. Specifically, a manual solid-phase microextraction (SPME) injector and a 50/30 μm DVB/CAR/PDMS fiber head were used to extract the volatile components of tea samples. The fiber head was aged at 230 °C for 5 min before use. About 1.0 g of homogenized black tea powder was extracted in 5.0 mL of boiling water with 1.0 mL of ethyl decanoate as the internal standard (1.0 mg. L^−1^). The mixed solution was equilibrated in a water bath at 60 °C for 5 min. Then, the SPME fiber was exposed in the headspace for 40 min. The fiber head was immediately inserted into the gas chromatograph injector for thermal desorption at 230 °C for 5 min.

#### 2.5.2. GC-MS/MS Analysis

GC conditions: DB-WAX GC column; carrier gas was helium (99.99%), column flow rate 1.0 mL.min^−1^, injection port temperature 250 °C, non-split injection, 1 μL per injection, solvent delayed for 4 min. Temperature rise program: 40 °C hold for 5 min, rise to 180 °C at a rate of 5 °C.min^−1^, hold for 2 min, then rise to 230 °C at a rate of 10 °C.min^−1^, hold for 2 min.

MS conditions: Electron Ionization (EI) was performed at 70 eV, and the ion source and quadrupole temperature were 230 °C and 150 °C, separately. The MS interface temperature was set at 250 °C. Full scan mode was used with a mass range of 20–550 aum and a solvent delay of 5 min [26].

### 2.6. Quantitative Analysis of Volatile Compounds and Calculation of Odor Activity Value (OAV)

Volatile compounds were semi-quantified based on the peak areas of the internal standard according to Fang et al. [27]. The relative odor activity value (rOAV) was calculated to evaluate the contribution of volatile compounds to black tea aroma. The rOAV was determined as the ratio of compound concentration (Ci) to its odor threshold (OTi) in water [28,29,30]. The calculation formula is as follows:

rOAV = Ci/OTi

Volatile compounds with an rOAV > 1 were considered to contribute to the overall aroma profile.

### 2.7. Statistical Analysis

The experiment was repeated three times. The data are expressed as means ± SD. Statistical analyses were performed using SPSS (IBM Corp, v23, Armonk, NY, USA). For samples that pass the homogeneity of variance test, a one-way ANOVA with Duncan multiple comparisons tests was used. For samples that did not pass the homogeneity of variance test, significance was calculated using the Tamhane T2 test. A value of *p* < 0.05 indicated statistical significance.

Multivariate analysis was performed to discriminate volatile compounds among different groups. Principal component analysis (PCA) and Orthogonal Partial Least Squares Discriminant Analysis (OPLS-DA) were conducted using SIMICA-P 14.1 (Umetrics, Umeå, Sweden) with unit variance scaling and the mean centering of data. For two-group analysis, differential metabolites were determined by VIP > 1 and |Log2FC| ≥ 1.0. For multi-group analysis, differential metabolites were determined by VIP > 1 and *p* < 0.05.

## 3. Results

### 3.1. The Effect of Different Light Withering Conditions on the Sensory Evaluation of Black Tea

As shown in Table 1, different light withering exerted diverse effects on the sensory quality of black tea. Compared to natural light withering (CK), single LED light (BL and YL) slightly decreased the total sensory score of black tea. On the contrary, the combination of red and yellow light withering improved the quality of black tea. In terms of aroma, the score in the RY group is the highest, followed by the BL group. This indicates that composite light withering is more effective in improving the aroma of black tea than single LED light treatment.

### 3.2. Effects of Different Light Withering Conditions on Major Quality Components of Black Tea

**Figure 1:**
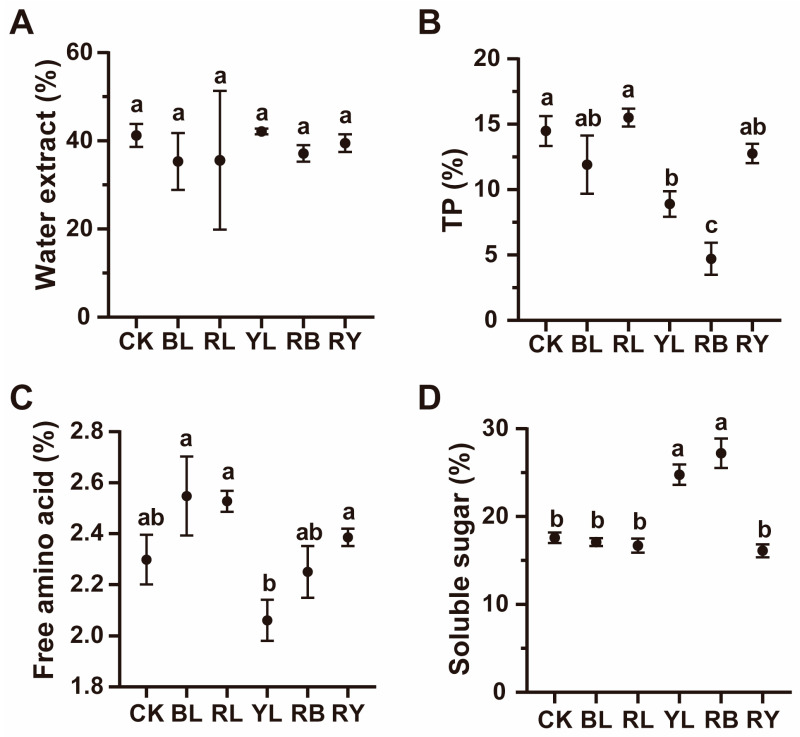
Effects of different light quality withering on major quality components of black tea. Content of water extract (**A**), tea polyphenols (**B**), free amino acid (**C**) and soluble sugar (**D**) in black tea withered with different lights. Data are means ± SD. Bars with different letters indicate significant difference (*p* < 0.05).

To elucidate the effects of different monochromatic and composite light withering conditions on black tea flavor, the contents of major quality components were detected. As depicted in Figure 1, different monochromatic and composite light withering conditions did not affect the water extract content of black tea (Figure 1A). Compared to natural light withering (CK), yellow light (YL) and red–blue light (RB) withering significantly decreased the content of TP (Figure 1B). In addition, the combination of red and blue light significantly reduced the TP content compared to withering with red and blue monochromatic light (Figure 1B). Compared to CK, different light withering conditions showed no significant effects on the content of free amino acid (Figure 1C). On the contrary, yellow (YL) and red–blue light (RB) withering increased soluble sugar content, while other monochromatic light and red–yellow composite light showed no statistical effect on it when compared to the CK group (Figure 1D). This indicates that appropriate LED light withering is effective in improving the flavor of black tea, and composite light treatment is more effective to some extent.

### 3.3. Preliminary Identification of Volatile Components in Black Tea Withered with Different Lights

**Figure 2:**
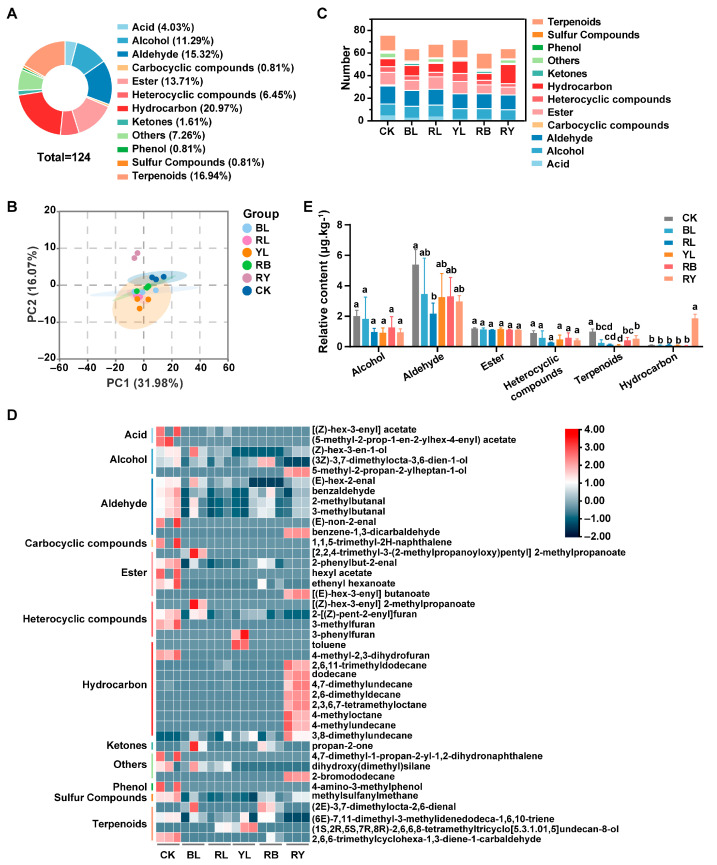
Volatile components in black tea withered with different lights. (**A**) Types of volatile substances in black tea withered with different lights. (**B**) PCA scores of volatile components in black tea withered with different lights. (**C**) Number of different types of volatile compounds detected in different groups. (**D**) Heatmap of differential volatiles in all groups. (**E**) Relative content of different types of volatiles (greater than 0.01 μg.kg^−1^) in different groups. Data are means ± SD. Bars with different letters indicate significant difference (*p* < 0.05).

A total of 124 volatile components were identified in all groups, which can be categorized into 12 types like acid (5), alcohol (14), aldehyde (19), carbocyclic compounds (1), ester (17), heterocylic compounds (8), hydrocarbons (26), ketone (2), phenol (1), sulfur compounds (1), terpenoids (21) and others (9) (Figure 2A) (Table S1). PCA showed that the discrepancy in volatile substances between nature light withering and other LED light withering was distinct. However, very little difference was observed between different LED light treatment groups (Figure 2B). Of all the groups, the natural light withering treatment (CK) had the highest amount of volatile substances, followed by the yellow, red, red–yellow, blue and the red–blue light treatment groups (Figure 2C). Among them, the CK group possesses the most amount of aldehydes, alcohols and esters. Volatile components in the BL and RL groups were mainly aldehydes, terpenoids, alcohols and esters. The YL group exhibited higher quantities of terpenoids than other groups, and it also comprised a considerable amount of aldehydes, esters, and hydrocarbons. Volatiles in the RB group were mainly terpenoids and aldehydes, whereas hydrocarbons and aldehydes dominated in the RY group (Figure 1C). A heatmap of the relative contents of volatile components showed that the natural light-treated group (CK) comprised much higher level of acids, aldehydes, esters, and heterocyclic compounds than other groups (Figure 2D, Table S1). The relative content of different classes of compounds (those greater than 0.01 μg.kg^−1^) showed that red light withering significantly decreased the level of aldehydes (Figure 2E). Compared to the CK group, all LED light treatments used in this study significantly reduced the terpenoid content, with YL showing the lowest level, followed by the RL and BL groups. Interestingly, the hydrocarbons level in the RY group was higher than that of other groups (Figure 2E).

### 3.4. Effects of Different Monochromatic Light Withering Conditions on the Aroma of Black Tea

**Figure 3:**
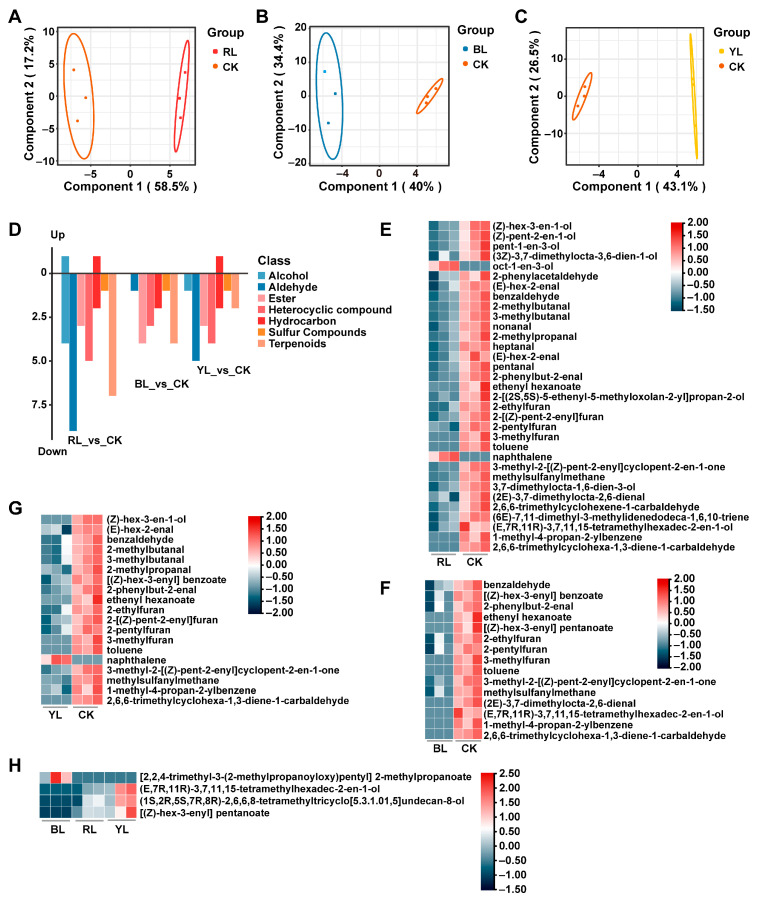
Effects of different monochromatic light withering conditions on the aroma of black tea. The OPLS-DA score plot of the RL_vs_CK (**A**), BL_vs_CK (**B**), and YL_vs_CK (**C**) groups. (**D**) Types of differential volatile components screened from the RL_vs_CK, BL_vs_CK, and YL_vs_CK groups. A heatmap of differential volatile compounds in the RL_vs_CK (**E**), BL_vs_CK (**F**), YL_vs_CK (**G**), and BL_vs_RL_vs_YL (**H**) groups.

To investigate the effect of different monochromatic light withering conditions on the aroma of black tea, OPLS-DA analysis was conducted on different monochromatic light treatment groups (RL, BL, and YL) and the natural light treatment group (CK), respectively. The results showed that the RL, BL and YL groups did not intersect with the CK group, proving that the OPLS-DA model could correctly distinguish the black tea withered with different monochromatic lights (Figure 3A–C). Cross-validation revealed that these three present models were not overfitted (Figure S1A–C). A total of 33 differential volatiles, mainly aldehydes and terpenoids, were found in the RL vs. CK group (Table S2, Figure 3D). Except for oct-1-en-3-ol and naphthalene, other compounds were decreased in the RL group (Figure 3E). Further analysis revealed that 11 of these substances possessed rOAVs greater than 1. Of these, eight had definite odors exhibiting fruity, almond, and grassy fragrances (Table 2). Only 15 differential volatiles were screened in the BL_vs_CK group, predominantly esters, terpenoids, and heterocyclic compounds (Table S2, Figure 3D). Interestingly, all these 15 components showed a decreasing trend in the BL group (Figure 3D,F), of which only 3 substances had an rOAV > 1, namely methylsulfanylmethane, (2E)-3,7-dimethylocta-2,6-dienal, and 2-pentylfuran. These volatiles presented cabbage, sweet, wet earth and lemon odors, separately (Table 3) [31]. In the YL_vs_CK group, 19 differential volatile compounds were screened, which were dominated by aldehydes and hydrocarbons (Table S2, Figure 3D). Among these, only naphthalene was elevated in the YL group (Figure 3G). In addition, only five substances with an rOAV > 1 presented almond, bitter, green, and fruity odors (Table 4). However, a slight difference was observed in the volatile components of different monochromatic light withering groups (Figure 3H).

### 3.5. Aroma Profile of Black Tea Withered with Different Composite Lights

**Figure 4:**
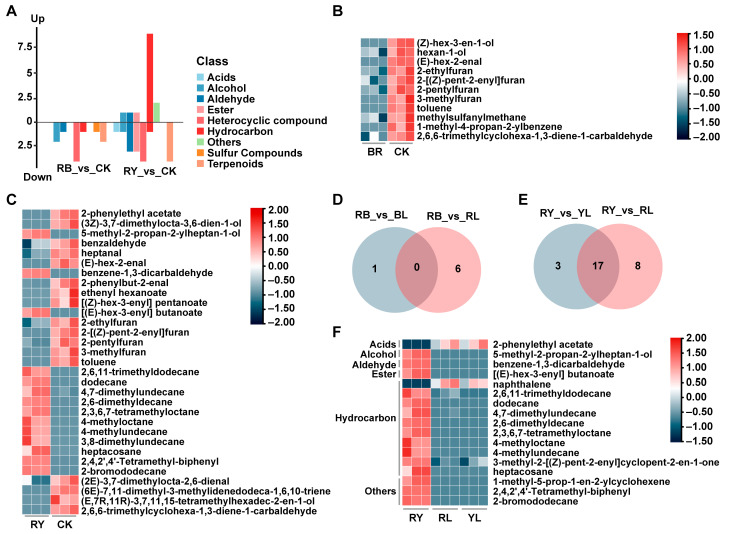
Effects of different composite light withering conditions on black tea aroma. (**A**) Types of differential volatile components screened from RB_vs_CK and RY_vs_CK groups. Heatmap of differential volatile compounds in RB_vs_CK (**B**) and RY_vs_CK (**C**) groups. Venn graph illustrates volatile components affected only by red–blue (**D**) and red–yellow (**E**) light withering. (**F**) Heatmap of differential volatile compounds in RY_vs_RL_vs_YL group.

In the RB_vs_CK group, 11 differential volatiles were identified (Table S2). Compared to CK, the level of a few alcohols, aldehydes, heterocyclic compounds, hydrocarbons, and terpenoids was decreased in the RB group (Figure 4A,B). Of these components, (E)-hex-2-enal, 2-methylbutanal, and 3-methylbutanal comprised an rOAV > 1, which exhibited almond and green odors (Table 5). In contrast, a total of 31 differential volatile compounds were screened in the RY_vs_CK group (Table S2), which included hydrocarbons, heterocyclic compounds, esters, aldehydes, terpenoids, etc. (Figure 4A). Among these substances, the content of nine hydrocarbons like 2,6,11-trimethyldodecane, dodecane, and 4,7-dimethylundecane was increased in the RY group. Additionally, a few alcohols (5-methyl-2-propan-2-ylheptan-1-ol), an aldehyde (benzene-1,3-dicarbaldehyde) and an ester ([(E)-hex-3-enyl] butanoate) were elevated by red–yellow light (Figure 4A,C). In the RY_vs_CK group, substances with an rOAV > 1 mainly presented fruity and floral flavors (Table 6).

**Table 5:** Key volatile components (rOAV > 1) screened in RB_vs_CK group.

Volatile Components	Threshold (μg/kg)	rOAV		Odor
		RB	CK	
(E)-hex-2-enal	0.25	0.00	2456.60	almond, bitter, green, heavy
2-methylbutanal	300	1.40	3.27	cabbage, organic, sulfur, wet earth
3-methylbutanal	4.8	7.71	23.21	almond, cheese, chocolate, malt

**Table 6:** Key volatile components (OAV > 1) screened in RY_vs_CK group.

Volatile Components	Threshold (μg/kg)	rOAV		Odor
		RY	CK	
(2E)-3,7-dimethylocta-2,6-dienal	5	2.00	10.74	lemon
2-phenylethyl acetate	20	0	2.82	flower, honey, rose
2-pentylfuran	4.8	9.012	23.21	butter, floral, fruit, green bean
heptanal	31	0.78	1.60	citrus, fat, green, nut

The results obtained above indicated that both single LED light and composite light withering improved black tea aroma. To explore the volatile components influenced only by composite light, we performed a Venn analysis of the combined light treatment group and the single LED light group. The results showed that the number of compounds that were only affected by the red–blue light withering was 0 (Figure 4D), whereas 17 volatiles were affected only by red–yellow light withering (Figure 4E). These 17 substances comprised hydrocarbons, acids, esters, alcohols, and aldehydes (Figure 4F). Except for 2-phenylethyl acetate and naphthalene, the content of these volatiles was higher in the RY group than in the RL and YL groups (Figure 4F), and only 2-phenylethyl acetate possessed an rOAV > 1. In the RB_vs_RY group, 19 differential metabolites were screened and it was dominated by hydrocarbons, along with a few alcohols, aldehydes, and terpenoids. Among these compounds, most exhibited higher concentrations in the RY group compared to the RB group (Figure S1D). This indicates that red–yellow light withering is more effective in altering the aroma of black tea.

## 4. Discussion

In this study, the effect of different monochromatic LED lights and composite light withering on black tea aroma was investigated. Consistent with previous studies [14,27], appropriate exogenous light exposure during withering altered both the composition and concentration of volatile compounds as well as the major quality components. However, our results demonstrated that single-wavelength LED treatments (BL, RL and YL) tended to reduce the contents of terpenoids, aldehydes, esters and heterocyclic compounds compared to natural light withering (CK). Composite light treatments, particularly red–yellow (RY) light, exhibited more pronounced effects on volatile profiles than monochromatic light, which significantly elevated the level of heterocyclic compounds and some alcohols and aldehydes.

The effect of the light spectrum on tea flavor during withering has been the focus for both producers and researchers. It affects the sensory quality and chemical composition of tea with different wavelengths and intensities [14,32]. To date, various LED lights including white, red, yellow, blue, green, purple, ultraviolet (UV), orange light, and multi-wavelength LED combinations have been applied in the withering process of different kinds of tea [16,33]. LED light withering has been reported to increase the water extract of tea [32]. However, no significant difference was observed in the water extract content in different groups (Figure 1A). This may be because the CK group received composite spectrum sunlight containing both visible and invisible wavelengths. In other words, it is a compound light. In addition, the effects of different LED light withering conditions on the major components of tea vary, with the blue light group showing the lowest TP content and the red light group exhibiting the highest amino acid level [32]. In the present study, the total amount of TP was decreased in the YL and RB groups, potentially attributed to the alteration in the expression of relevant genes by exogenous light [34]. Meanwhile, the YL group displayed the lowest free amino acid level, aligning with a previous study in which yellow light withering significantly reduced amino acid content compared to the red and blue light groups [32].

Moreover, withering with different lights impacts the aroma profiles of tea. It was found that red light reduced the grassy odor but enhanced the floral and fruity fragrance [14]. This is consistent with what we observed in this study that the red light withering group exhibited a strong sweet fragrance. Nevertheless, the quantity and content of volatile components tended to decrease in the single LED light treatment groups (RL, BL, and YL) with respect to the CK group (Figure 2), which was attributed to the fact that the CK group was a composite light. However, it is reported that no significant cumulative effect was found in the hybrid light withering treatments on the flavor components of black tea [33]. This discrepancy may be due to variations in the intensity and duration of the monochromatic light used. Little difference in volatiles was observed among different monochromatic light-treated groups, which coincided with the findings of Ai et al. [33] but disagreed with those of Hua et al. [14]. In the latter, blue light withering promoted the accumulation of geraniol and citral [14]. Such disparities may stem from differences in experimental parameters like light intensities and treatment time [15]. Notably, the effects of monochromatic yellow light withering on volatiles and biochemical components of black tea appeared to occur prior to those of red and blue light in this study, as evidenced by the lowest content of TP and the highest level of soluble sugars, (E,7R,11R)-3,7,11,15-tetramethylhexadec-2-en-1-ol, (1S,2R,5S,7R,8R)-2,6,6,8-tetramethyltricyclo [5.3.1.01,5] undecan-8-olde, and [(Z)-hex-3-enyl] pentanoate, which exhibited distinct floral and fruity notes [31]. This is consistent with a previous study that showed that yellow light (5000 lx) surpassed red and blue light in improving the aroma of white tea [35].

In practical, natural light withering is more widely used than single-wavelength LED treatments, suggesting that potential advantages of composite light withering emerge. This is supported by He et al. [36] who demonstrated that moderate-intensity composite LED light treatment produced a better aroma compared to natural light. This result is consistent with our findings showing that the RB group exhibited a higher aroma score. Red–yellow light withering increased the content of hydrocarbons, certain aldehydes, and alcohols compared to monochromatic lights, which resulted in an almond-like and roasted aroma of the black tea. This indicates that composite light withering is effective in improving tea aroma. However, this finding disagrees with a prior study where monochromatic yellow light withering outperformed red–blue light in enhancing white tea aroma [37]. The discrepancy may arise from subsequent processing steps (e.g., rolling, fermentation) in the present study, which were also crucial for the development of aroma [37]. Notably, our data showed that red–yellow light exerted stronger effects on black tea volatiles than the red–blue treatment, aligning partially with the result obtained above that yellow light is superior to monochromatic red and blue light.

## 5. Conclusions

In summary, this study investigated the effects of different monochromatic and composite light withering conditions on the aroma of black tea. We found that yellow light withering enhanced soluble sugar content while reducing TP levels in black tea. It also increased total volatile compounds more effectively than red or blue light treatments, demonstrating a superior improvement on black tea aroma. In addition, composite light withering, particularly red–yellow light, outperformed single-wavelength LED treatments. Red–yellow light significantly elevated hydrocarbons, certain aldehydes, and alcohols, which ultimately imparted fruity (e.g., almond-like) and roasted aroma notes to the black tea.

## Figures and Tables

**Table 1:** Sensory evaluation result of black tea withered via different lights.

Treatments	Appearance (25%)		Infusion Color (10%)		Aroma (25%)		Taste (30%)		Infused Leaf (10%)		Total Score
	Terms	Score	Terms	Score	Terms	Score	Terms	Score	Terms	Score	
CK	Relatively tightly rolled and even	84	Bright orange-red	85	Prominent dried longan fragrance	84	Strong and mellow with a lingering sweetness	87	Slightly mixed colors, soft	85	85.1
BL	Tight and sturdy, dark in color	84	Bright orange-red	85	Floral and fruity fragrance	84	Mellow with a lingering sweetness	83	Slightly mixed colors, soft	84	83.8
RL	Sturdy and tightly bound, dark and glossy	88	Bright orange-red	80	Sweet fragrance	88	Slightly astringent, with a hint of sweetness	90	Oily and evenly red	85	87.5
YL	Thin and tightly rolled	80	Orange-red	80	Dried longan fragrance	80	Mellow	84	Bronze in color	82	81.4
RB	Relatively thin and tightly bound, still dark and glossy with good cleanliness	84	Orange-red, relatively bright	80	Roasted fragrance	84	Mellow with a lingering sweetness	84	Slightly mixed colors, soft	85	83.7
RY	Sturdy and tightly bound, dark and glossy	90	Translucent orange-red	90	Roasted aroma, strong	90	Mellow and refreshing	90	Soft, orange-red, shiny	90	90

Note: CK: natural light withering, BL: blue light withering, RL: red light withering, YL: yellow light withering, RB: red–blue composite light, RY: red–yellow composite light.

**Table 2:** Key volatile components (rOAV > 1) screened in RL_vs_CK group.

Volatile Components	Threshold (μg/kg)	rOAV		Odor
		RL	CK	
2-phenylacetaldehyde	4	330.90	742.48	berry, geranium, honey, nut, pungent
3,7-dimethylocta-1,6-dien-3-ol	1.5	377.81	1027.85	aniseed, floral, fragrant citrus
(E)-hex-2-enal	0.25	1208.21	2456.60	almond, bitter, green, heavy, green
2-methylbutanal	8.8	10.28	43.84	almond, burnt, choking, cocoa, estery apple, fermented, fruity, green grass
3-methylbutanal	0.25	277.13	1089.19	almond, cheese, chocolate, malt,
2-methylpropanal	0.7	40.74	132.43	burnt, caramel, cocoa, green, malt
2-pentylfuran	4.8	3.48	23.21	butter, floral, fruit, green bean
oct-1-en-3-ol	2	3.32	/	/
(2E)-3,7-dimethylocta-2,6-dienal	5	2.58	10.74	/
nonanal	3.5	11.09	32.66	/
Heptanal	3.5	0.58	1.60	citrus, fat, green, nut

Note: The threshold of volatile compounds in the water was reported by GEMERT [30], the same as in Table 3, Table 4, Table 5 and Table 6.

**Table 3:** Key volatile components (rOAV > 1) screened in BL_vs_CK group.

Volatile Components	Threshold (μg/kg)	rOAV		Odor
		BL	CK	
2-phenylacetaldehyde	4	330.90	742.48	Berry, Geranium, Honey, Nut, Pungent
methylsulfanylmethane	300	0.83	3.27	Cabbage, Organic, Sulfur, Wet Earth
(2E)-3,7-dimethylocta-2,6-dienal	5	/	10.74	Lemon
2-pentylfuran	4.8	6.96	23.21	Butter, Floral, Fruit, Green Bean

**Table 4:** Key volatile components (rOAV > 1) screened in YL_vs_CK group.

Volatile Components	Threshold (μg/kg)	rOAV		Odor
		YL	CK	
(E)-hex-2-enal	0.25	706.02	2456.60	almond, bitter, green, heavy
2-methylbutanal	8.8	14.64	43.84	almond, burnt, choking, cocoa, estery apple, fermented, fruity, green grass
3-methylbutanal	0.25	366.79	1089.19	almond, cheese, chocolate, malt
2-methylpropanal	0.7	22.88	132.43	burnt, caramel, cocoa, green, malt
2-pentylfuran	4.8	8.74	23.21	butter, floral, fruit, green bean

## Data Availability

The original contributions presented in the study are included in the article, further inquiries can be directed to the corresponding author.

## Supplementary Materials

The following supporting information can be downloaded at https://www.mdpi.com/article/10.3390/foods14132232/s1, Figure S1: The hypothesis testing of the OPLS-DA model in RL_vs_CK (**A**), BL_vs_CK (**B**), and YL_vs_CK (**C**) groups, and a heatmap of differential volatiles in the RB_vs_RY group (**D**); Table S1: The original data of all volatile compounds detected in this study; Table S2: Differential volatiles screened in different groups.
