# Supplementary figures and images for: Effects of Monochromatic and Composite Light Withering on Black Tea Aroma
Source: Foods. 2025 Jun 25;14(13):2232. doi: 10.3390/foods14132232 (PMC12249116; doi:10.3390/foods14132232)

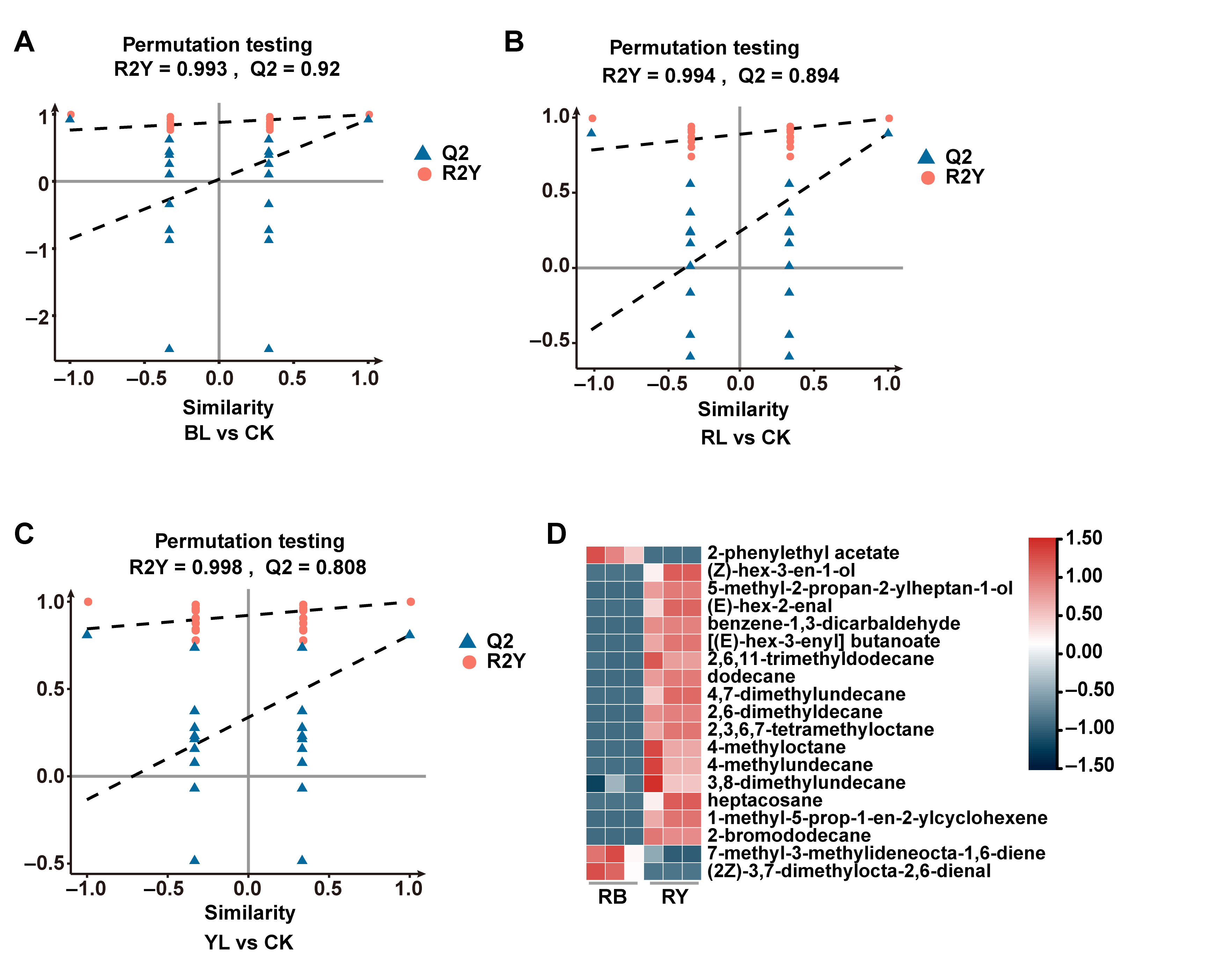

Supplement: Supplementary file 1 [file foods-14-02232-s001.zip › Figure S1.tif]
